# Tick-Borne Diseases in Turkey: A Review Based on One Health Perspective

**DOI:** 10.1371/journal.pntd.0005021

**Published:** 2016-12-15

**Authors:** Abdullah Inci, Alparslan Yildirim, Onder Duzlu, Mehmet Doganay, Serap Aksoy

**Affiliations:** 1 Department of Parasitology, Faculty of Veterinary Medicine, Erciyes University, Kayseri, Turkey; 2 Vectors and Vector-Borne Diseases Implementation and Research Centre, University of Erciyes, Kayseri, Turkey; 3 Department of Infection Diseases and Clinical Microbiology, Faculty of Medicine, Erciyes University, Kayseri, Turkey; 4 Department of Epidemiology of Microbial Diseases, Yale School of Public Health, New Haven, Connecticut, United States of America; University of California San Diego School of Medicine, UNITED STATES

## Abstract

The importance of tick-borne diseases is increasing all over the world, including Turkey. Global warming, environmental and ecological changes and the existence of suitable habitats increase the impact of ticks and result in frequent emergence or re-emergence of tick-borne diseases (TBDs) with zoonotic characteristics. In Turkey, almost 19 TBDs have been reported in animals and men, involving four protozoa (babesiosis, theileriosis, cytauxzoonosis, hepatozoonosis), one filarial nematode (acanthocheilonemasis), ten bacterial agents (anaplasmosis, ehrlichiosis, aegyptianellosis, tick-borne typhus, *Candidatus* Rickettsia vini, Lyme borreliosis, tick-borne relapsing fever [TBRF], tularaemia, bartonellosis, and hemoplasmosis), and four viral infections (tick-borne encephalitis [TBE], Crimean-Congo Haemorrhagic Fever [CCHF], louping-ill [LI], and lumpy skin disease [LSD]). The growing number of TBD cases, in particular the fatal viral epidemics in humans, have led to increased public awareness and concern against TBDs in recent years. The World Health Organization (WHO) has developed a new political concept, called the “One Health” initiative, which is especially relevant for developing strategies against tick infestations and TBD control in humans and animals. It would be beneficial for Turkey to adopt this new strategy and establish specific research and control programs in coordination with international organizations like WHO, the World Organization for Animal Health (OIE), the Food and Agriculture Organization (FAO), the Centers for Disease Control and Prevention (CDC), and the European Center for Disease Prevention and Control (ECDC) to combat TBDs based on the “One Health Initiative” concept. In this article, we review the occurrence of primary TBDs in man and animals in Turkey in light of the “One Health” perspective.

## Introduction

**Fig 1:**
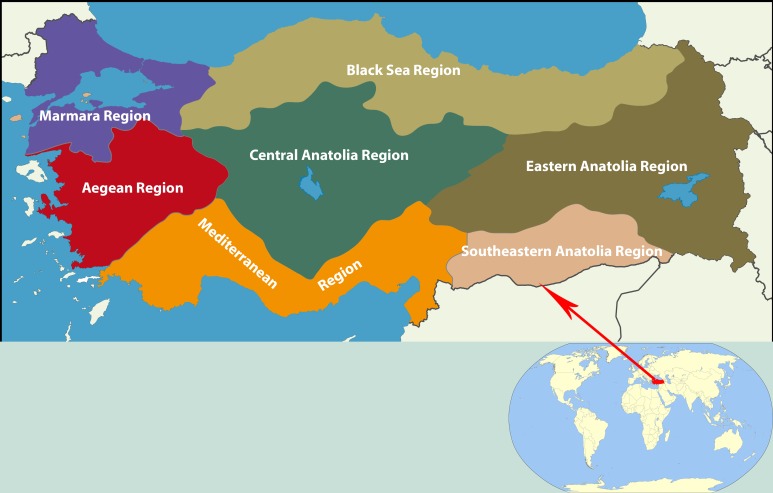
The geographic positioning of Turkey. The location of Turkey spanning the continents of Europe and Asia is shown. The seven geographic districts with varying ecological characteristics are included on the map. Image credit: The Emirr, Wikipedia Commons.

Turkey is subtropically located in Eurasia and has a population of over 80 million people, with 50 million livestock animals. The economic structure of Turkey currently depends on a mix of industrial and agricultural products. Turkey is divided into seven distinct geographic regions: Eastern Anatolia, Southeastern Anatolia, Mediterranean, Aegean, Marmara, Black Sea, and Central Anatolia (Fig 1).

**Fig 2:**
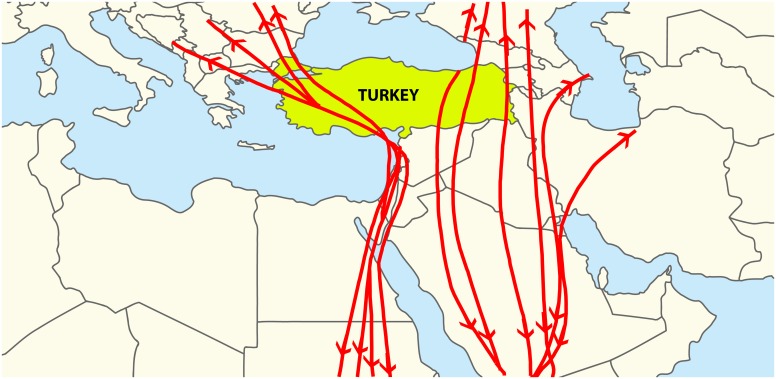
The main migration routes of birds passing through Turkey. Image credit: Shadowxfox, Wikipedia Commons.

The geographic location of Turkey provides a natural bridge for transmission of many emerging or re-emerging diseases among the continents of Europe, Asia, and Africa. Particularly, the many marshes or immigrate bird stations (Fig 2), like “Sultan Marshes” in the Kayseri area of Central Anatolia, “Manyas Bird Paradise” in the Marmara region, “Kizilirmak Delta” or “Cernek Ringing Station” in Bafra near Samsun in the Black Sea region, “Hevsel Bird Paradise” in Diyarbakir in the Southeast, and “Aras Bird Paradise” in the Northeast all have high epidemiological importance for the distribution of ticks and tick-borne diseases (TBDs). In addition, the geographic location of Turkey results in highly varied climatic conditions in the seven regions of the country. A typical continental climate prevails in the plateaus of Anatolia, while temperate climates mainly dominate the coastal areas. Each one of the seven geographic regions has different climatic conditions, vegetation structures, and wildlife allowing suitable habitats for various vector arthropods throughout the four seasons of the year.

Many disease pathogens that challenge the welfare of human, livestock, wildlife, and plants worldwide are transmitted to their hosts via specific arthropod vectors [1]. The public health impact and financial consequences of these diseases can devastate the already overburdened economic conditions in developing countries [2], as well as in Turkey [3]. Among these diseases, tick-borne pathogens are the most prevalent and dangerous for public health and livestock, especially during the tick seasons in most parts of Turkey. The morbidity and mortality of TBDs, such as babesiosis, theileriosis, and anaplasmosis annually changes depending on the enzootic stability of infections, and also on the immunological status of host animals. Some TBDs, particularly babesiosis and theileriosis, are known to be prevalent in many parts of Turkey. Tropical theileriosis is widespread in Turkey, with over 90% of animals being seropositive in some areas. Approximately 20%–60% of cattle may be exposed to the disease in one season. Several vector *Hyalomma* spp. are present in Turkey, and over 40% of ticks can be infected with *Theileria annulata* in the Central Anatolia, Aegean, and Eastern Anatolia regions. The mortality of infection in local cattle breeds is approximately 50%, but can be up to 100% in imported pure breeds [4]. Meanwhile, a re-emerging disease, Crimean-Congo Haemorrhagic Fever (CCHF), has been associated with many deaths since 2002 and reached a peak in 2008 and 2009, resulting in a total of 126 human deaths in endemic areas of Turkey [5].

In this article, we review the occurrence of TBDs in man and animals in Turkey based on the “One Health” concept. The “One Health” concept was developed to promote interdisciplinary collaborations and communications in all aspects of health care for humans, animals, and the environment. It is expected that this synergism will advance health care by accelerating biomedical research discoveries, enhancing public health efficacy, expanding the scientific knowledge base, and improving medical education and clinical care. Since TBDs equally impact animal and human health, the “One Health” approach is particularly relevant for control of these diseases.

## TBDs of Humans and Animals in Turkey

TBDs are caused by several pathogenic agents with global distribution. The pathogens mainly cause destruction of blood cells resulting in anemia, jaundice, hemoglobinuria, anorexia, and weight loss and also increase risk for other bacterial and fungal infections. In Turkey, the ticks that transmit disease belong to both the soft-tick family, Argasidae (genera *Argas*, *Ornithodorus*), as well as the hard-tick family, Ixodidae (genera *Dermacentor*, *Hyalomma*, *Haemaphysalis*, *Ixodes*, and *Rhipicephalus*) [6–8].

Reported major TBDs include protozoa (babesiosis, theileriosis, cytauxzoonosis, and hepatozoonosis), filarial nematode (acanthocheilonemiasis), rickettsial bacteria (anaplasmosis, ehrlichiosis, aegyptianellosis, tick-borne typhus or Mediterranean spotted fever [MSF], and *Candidatus* R. vini) and nonrickettsial bacteria (Lyme borreliosis, tick-borne relapsing fever [TBRF], tularemia, bartonellosis, and hemoplasmosis) and viruses (CCHF, tick-borne encephalitis [TBE], louping-ill [LI], and lumpy skin disease [LSD]) (Table 1).

**Table 1:** Tick-borne pathogens (TBPs), their hosts, and vector ticks in Turkey.

Origin of TBPs		TBDs	Species	Host	Tick species	Reference
***Protozoa Infections***		Babesiosis	*Babesia bigemina*, *B*. *bovis*, *B*. *divergens*, *B*. *major*, *B*. *occultans*, *B*. *ovis*, *B*. *crassa*, *B*. *Caballi*, *Babesia canis canis*, *B*. *canis rossi*, *B*. *canis vogeli*, *B*. *gibsoni*, *B*. *microti*	Ruminants, equids, canids, felids, rodents, human	*Rhipicephalus annulatus*, *Rhi*. *bursa*, *Rhi*. *turanicus*, *Rhi*. *calcaratus*, *Rhi*. *sanguineus*, *Hyalomma excavatum*, *H*. *rufipes*, *H*. *marginatum*, *H*. *dromedarii*, *Haemaphysalis punctata*, *Hae*. *parva*, *Hae*. *sulcata*, *Dermacentor marginatus*, *D*. *reticulatus*, *Ixodes ricinus*	[6,9,10]
		Theileriosis	*T*. *annulata*, *T*. *buffeli/orientalis/sergenti*, *T*. *ovis*, *T*. *equi*	Ruminants, equids	*H*. *marginatum*, *H*. *anatolicum*, *H*. *excavatum*, *H*. *detritum*, *Haemaphysalis* spp., *Rhipicephalus* spp.	[1,6,11]
		Cytauxzoonosis	*Cytauxzoon felis*	Domestic cat	?	[12]
		Hepatozoonosis	*Hepatozoon canis*	Canids, felids	*Rhi*. *sanguineus*	[9,13,14]
***Filarial Nematode infections***		Canine filariosis	*Acanthocheilonema reconditum*	Dogs	?	[15,16]
***Bacterial Infections***	**Rickettsial infections**	Anaplasmosis	*Anaplasma phagocytophilum*, *A*. *platys*, *A*. *marginale*, *A*. *bovis*, *A*. *ovis*, *A*. *centrale*	Ruminants, dogs, human	*Ixodes* spp., *Dermacentor* spp., *Rhipicephalus* spp., *Haemaphysalis* spp., *Hyalomma* spp., *Ornithodorus* spp.	[17,18]
		Ehrlichiosis	*Ehrlichia canis*	Dogs	*Rhi*. *sanguineus*?	[19–22]
		Aegyptianellosis	*Aegyptianella pullorum*	Duck	?	[23]
		Tick-borne typhus	*R*. *hoogstraali*, *R*. *aeschlimannii*, *R*. *slovaca*	Human, dogs	*H*. *marginatum*, *H*. *aegyptium*, *H*. *excavatum*, *D*. *marginatus*, *Hae*. *parva*,	[10,24]
		*Candidatus* R. vini	*R*. *vini*	Birds	*Ixodes arboricola*	[8]
	**Non-rickettsial infections**	Lyme borreliosis	*Borrelia burgdorferi*, *Bor*. *turcica* sp. nov.	Human, dogs, horses	*I*. *ricinus*, *H*. *aegyptium*, *H*. *marginatum*, *H*. *excavatum*, *Hae*. *parva*,	[24,25,26–28]
		TBRF	*Bor*. *crocidurae*	Rodents	*Ornithodoros erraticus*	[29]
		Tularemia	*Francisella tularensis*	Human	?	[30,31]
		Bartonellosis	*Bartonella henselae*	Cat	?	[32]
		Hemoplasmosis	*Mycoplasma haemofelis*	Cat	?	[33]
***Viral infections***		TBE	TBE virus	Human	?	[34–37]
		CCHF	CCHF virus	Human	*H*. *marginatum*, *Haaemaphysalis* spp., *Rhipicephalus* spp., *I*. *ricinus*	[5,38]
		LI	LI virus	Sheep	?	[39]
		LSD	LSD virus	Cattle	?	[40]

**Babesiosis** is a zoonotic and hemolytic infection of animals and man caused by *Babesia* spp., and transovarially and trans-stadially transmitted by ixodid ticks [41]. The disease is characterized by high temperature, anemia, icterus, hemoglobinuria, listlessness, anorexia, and death. Although the major economic impact of babesiosis is on the cattle industry, the infection has been seen in other domestic animals, including horses, sheep, goats, pigs, and dogs, in varying degrees of importance throughout the world [42]. Babesiosis is the first described tick-borne infection in Turkey and has been reported from cattle, sheep/goats, horses, dogs, and man. Bovine and ovine babesiosis are highly prevalent throughout the country, whereas there are no reports about clinical cases of human babesiosis [1,4].

**Theileriosis** is another tick-borne protozoan disease of ruminants, equids, and felids. The causative agents of the infection belonging to *Theileria* are transmitted from an infected animal to others by trans-stadial transmission via ticks [43]. The infection appears in two clinical forms in infected animals: malignant and benign theileriosis. The malignant form causes a lymph proliferative disease with high morbidity and mortality in susceptible European cattle breeds, whereas mild infections occur in the indigenous breeds. The main characteristics of the diseases are lymphoid transformation, proliferations, immortalization, and metastasis of schizont-infected monocytes and lymphocytes, resembling cancer [44]. Considerable economic losses have been reported due to malignant cattle theileriosis in endemic areas of the world [45]. Bovine theileriosis is also an important parasitic disease of Turkey. The prevalence of *T*. *annulata* infection, morbidity, and mortality was significantly higher in unvaccinated than in vaccinated cattle, whereas the seropositivity was significantly lower in the unvaccinated group. Acute tropical theileriosis cases were diagnosed in 156 of 554 (27.61%) cattle, and 86 of 156 (56.21%) died from the disease in the Cappadocia area of Central Anatolia in Turkey. The total economic losses due to tropical theileriosis were estimated at US$598,133 during 1999–2001 in the area [3]. In addition, ovine theileriosis [1] and equine theileriosis [11] have also been recently reported. Although human theileriosis has not been reported from Turkey, the responsible TBP, *T*. *microti*, was shown to circulate in Anatolian squirrel (*Spermophilus xanthophrymnus*) populations [46].

**Cytauxzoonosis** is a TBD of domestic cats [47]. The causative agent of the infection, *C*. *felis* is transmitted by *Amblyomma americanum* [48]. Most recently, *C*. *felis* was first reported from domestic Van cats in Turkey [12].

**Hepatozoonosis** is one of the TBDs of dogs. Old World canine hepatozoonosis caused by *Hep*. *canis* is transmitted by *Rhi*. *sanguineus*, whereas American canine hepatozoonosis caused by *Hep*. *americanum* is transmitted by *Am*. *maculatum* [49]. In Turkey, *Hep*. *canis* infection was detected in dogs [13,50], and mature and immature oocytes as well as sporocysts of *Hep*. *canis* were identified in *Rhi*. *sanguineus* ticks removed from dogs [9,14].

**Anaplasmosis** is an opportunistic and widespread vector-borne infection of humans and animals, caused by *Anaplasma* species including *A*. *marginale*, *A*. *centrale*, *A*. *bovis*, *A*. *ovis* for ruminants, *A*. *platys* for canines, and *A*. *pagacytophilum* for human and domestic animals such as horses. The infection is transmitted mainly intrastadially but also iatrogenically by ticks. The disease is called “Human Granulocytic Anaplasmosis” or “Human Granulocytic Ehrlichiosis” (HGE) in man, “bovine anaplasmosis” in cattle, “ovine anaplasmosis” in sheep, and “canine anaplasmosis” in dogs. The etiologic agent of HGE, *A*. *phagacytophilum*, is transmitted intrastadially by *Am*. *americanum* ticks in endemic areas [51]. *A*. *phagocytophilum* was determined in farm animals [52] and also in humans [53] in Turkey. Additionally, *A*. *phagocytophilum* was detected in *I*. *ricinus* ticks removed from humans [17]. A few bovine anaplasmosis outbreaks were reported in cattle from some areas [18,54,55], and one *A*. *platys* infection was shown in a dog in Turkey [56].

**Ehrlichiosis** is caused by *A*. *phagocytophilum*, *E*. *chaffeesis*, and *E*. *ewingii* in humans, and called “Human Monocytotropic Ehrlichiosis(HME); *A*. *phagocytophilum* and *E*. *canis* in dogs, and called “Canine Monocytotropic Ehrlichiosis (CME). The diseases are transmitted by ixodid ticks, and the public health and veterinary importance of Ehrlichiae was emphasized [57]. In Turkey, unfortunately the studies on CME are very limited. However, a few reports have documented seropositivity [19], clinical cases, treatment [20], and molecular prevalence [21,22] of CME in Turkey.

**Aegyptianellosis** is an intraerythrocytic tick-borne rickettsial infection of amphibians, reptiles, and birds. The infection is caused by *Ae*. *pullorum* and is transmitted to fowls by *Argas persicus* ticks [58,59]. One *Ae*. *pullorum* infection case was reported from ducks in Turkey [23].

**Tick-borne Typhus** is one of the oldest tick-borne rickettsial diseases. In Turkey, several cases of MSF associated with *R*. *conorii* have been reported from humans [60–64]. Recently, *R*. *hoogstraali* and two human pathogenic species (*R*. *aeschlimannii* and *R*. *slovaca)* were detected in ixodid ticks in Turkey [10,24]. *Candidatus* R. vini was detected in *I*. *arboricola* ticks collected from birds in the Kizilirmak Delta of Turkey [8].

**Lyme borreliosis** is a widespread and zoonotic tick-borne bacterial infection of humans and dogs in the north hemisphere [65]. Lyme disease is caused by spirochetes that comprise a complex referred to as *Bor*. *burgdorferi sensu lato*, with five major species that cause human disease. *Bor*. *burgdorferi* is used to refer to the whole complex and transmitted to humans and dogs by *Ixodes* spp. [66]. In Turkey, *Bor*. *burgdorferi* was isolated from *I*. *ricinus* ticks collected from cattle in the Black Sea region in 1998 [25], and spirochetes of *Borrelia* were present in an unfed tick nymph [67]. Meanwhile, some *Bor*. *burgdorferi sensu lato* strains were characterized molecularly [26], and a novel *Borrelia* sp. was also isolated from *H*. *aegyptium* ticks collected from tortoises (*Testudo graeca*) [27], and the spirochete was named as *Bor*. *turcica* sp. nov. [28]. A clinical Lyme case was observed in a dog in 2007 [68], and anti-*Bor*. *burgdorferi* antibodies were detected in dogs and horses in Turkey [69]. Recently, *Bor*. *burgdorferi sensu stricto* was isolated from unusual tick species, *H*. *marginatum*, *H*. *excavatum*, *Hae*. *parva*, and nymphs of *Hyalomma* spp. in Turkey [24].

**TBRF** is a spirochete disease of man caused by *Borrelia* spp. associated with the bite or coxal fluid of argasid ticks of the genus *Ornithodoros* in a wider endemic geographic area of the world and occurs in Africa, Asia, and the Americas with different *Borrelia* tick vector complexes in each area [66]. In Turkey, the presence of relapsing fever with a spirochete of the Crocidurae group, *Bor*. *crocidurae*, was also shown in *O*. *erraticus* ticks collected from rodent holes in the southeastern areas near the Syria border [29].

**Tularemia** is an arthropod-transmitted zoonotic bacterial infection caused by the *F*. *tularensis* and comprises a range of clinical syndromes ranging from mild to very severe. The majority of cases occur in the northern hemisphere, particularly in rural or semirural environments [66]. In Turkey, tularemia is an important disease, which has re-emerged in 1988, and the first tularemia outbreak was recorded in 2005 [70]. The first case associated with the outbreak was diagnosed near Kayseri, and the region was described as an endemic area for tularemia [30], but no positivity was detected in pools of mosquitoes and ticks collected near the Kayseri area by molecular techniques [31].

**Bartonellosis** is another zoonotic vector-borne infection of humans that is caused by *Bar*. *henselae*, with a large distribution in the northern hemisphere [71]. Domestic cats represent the main reservoir of the pathogen, and the main vector of the infection is the cat flea [72]. However, the trans-stadial transmission of *Bar*. *henselae* by *I*. *ricinus* ticks was also shown [73]. In Turkey, a solitary study on bartonellosis was reported in domestic cats [32].

**Hemoplasmosis** is one of the bacterial infections of humans and animals caused by *Mycoplasma* spp. [74]. Although the infection is mainly described as vector-borne and transmitted by blood-feeding arthropods such as ticks and fleas, the disease might also be transmitted through other routes, such as mechanically with contaminated operation tools or blood transfusions and vertically in the intra-uterine period [75]. *Rhi*. *appendiculatus* transmits the infection to dogs by cofeeding [76]. In Turkey, a clinical case about feline hemoplasmosis-associated *M*. *haemofelis* was reported [33].

**TBE** is an important infection of humans prevalent in a large endemic area of Asia and Europe. The disease agent is a virus belonging to the genus Flavivirus [77]. In Turkey, a few serosurveys were performed in the Southeast [34], the Central Anatolia [35] and Aegean regions [36,37], and in Central/Northern Anatolia [78], in which the seropositivities were reported in the range of 1.4% to 20.5%.

**CCHF** is a contagious and re-emerging infection of man transmitted by several ixodid ticks [79]. Wild and livestock animals serve as amplifiers of the CCHF virus in field conditions [38]. In Turkey, the infection was first observed in 2002 around Tokat in the Black Sea region and spread to neighboring cities initially and then throughout the country [80,81]. There has been an increase in the cases in following years, reaching a peak in 2008 and 2009, with a decrease thereafter. However, nearly 900 new CCHF cases are now seen annually, and a total of 9,787 cases have been reported from 2002 through 2015, resulting in 469 deaths (4.79%) [5].

**LI** is a tick-transmitted and acute viral disease of mainly sheep/goats, but it can sometimes also affect cattle and horses. The LI virus characteristically causes an encephalomyelitis disease of sheep. The occurrence of LI was found closely related to the distribution of the primary vector tick, *I*. *ricinus*, and the infection was reported from various countries including England, Norway, Greece, and Bulgaria [82], as well as the northwestern part of Turkey [39].

**LSD** is a pox disease of cattle characterized with nodules on the skin, transmitted mechanically via blood-feeding arthropods, including some hard ticks. It was reported that *Rhi*. *(Boophilus) decoloratus*, *Rhi*. *appendiculatus*, and *Am*. *hebraeum* ticks have a transmission role in the epidemiology of LSD in the endemic areas [83]. An LSD outbreak was recognized in cattle associated with the nodular clinical symptoms in Turkey [40]. Recently, a new confirmed LSD outbreak with huge economical devastation has been reported by official government veterinaries in May and June months of 2016 in Aegean region of Turkey and more than 500 cattle infected with LSD virus have been culled for the control of the disease (Prof. Abdullah Inci, personal communication).

## Conclusion

In this review, we focused on the assessment of TBDs in Turkey with a holistic approach. Turkey’s natural conditions allow exposure to many tick-borne infections in animals and humans in different regions. A total of 19 tick-borne infections have already been reported from seven major regions of Turkey. Many of these diseases result in significant economic losses and pose major public health threats. Millions of migratory birds, which utilize the sanctuaries present in Turkey during their annual migration, pose a constant threat to the spread of new infectious agents. Despite the high endemicity of tick borne pathogens and presence of suitable tick habitats in Turkey, in depth epidemiological studies and research investigations into TBDs are lacking. Thus, Turkey urgently needs to develop a new disease management strategy and establish the infrastructure for control programs against ticks and TBDs. We propose that a new framework be adopted in coordination with international bodies including WHO, the World Organization for Animal Health (OIE), the Food and Agriculture Organization (FAO), the Centers for Disease Control and Prevention (CDC), and the European Center for Disease Prevention and Control (ECDC) based on the modern concept “One Health Initiative” or “One Medicine Perspective.” This strategy should include predictable scenarios for the future of TBDs based on knowledge of the host—pathogen—tick “disease triangle” with regards to global warming, environmental changes, and socioeconomic status of affected human societies and the ecology of tick habitats and tick distributions. To interfere with this triangle, the first and urgent step is to organize and initiate an integrated tick control program through the disease endemic regions of the country. Towards this purpose, the application of new and advanced tick control techniques, such as recombinant anti-tick vaccines applicable for animals, can be investigated. Support of crosscutting and interdisciplinary tick research projects in specialized research centers would facilitate knowledge and future applications. Another important approach is to improve disease-resistant animals instead of susceptible breeds against TBDs using vaccination and immunization programs, especially for babesiosis, theileriosis, and anaplasmosis. At this point, the development of the CCHF vaccine is of critical importance in order to control disease in the human host in endemic areas of Turkey.

In accordance with the “One Health” concept, development of advanced research projects for control of ticks and TBDs by expert researchers from all related scientific disciplines should be a top priority. On the other hand, the administrative and political decisions that impact climate change, urbanization, land use, and industrial and agricultural pollution should be consistent with ecological and epidemiological findings on ticks and TBDs in Turkey. We also suggest that a regional program utilizing the One Health Concept that takes into account an interdisciplinary approach would be imperative to combat TBDs given that country borders are insignificant for disease transmission. Turkey should provide leadership to bring together the agencies and appropriate researchers under the auspices of international organizations to help shape a global policy for TBD control in the region.

**Top Five Papers**

1. Inci A, Yazar S, Tuncbilek AS, Canhilal R, Doganay M, Aydin L, et al. Vectors and vector-borne Diseases in Turkey. Ankara Univ Vet Fak Derg. 2013; 60: 281–96.
2. Sayin F, Dincer D, Karaer Z, Cakmak A, Inci, A, Yukari BA, et al. Tick-borne diseases in Turkey. Trop Anim Hlth Prod. 1997; 29: 535.
3. Aydin L, Bakirci S. Geographical distribution of ticks in Turkey. Parasitol Res. 2007; 101(Suppl 2): S163-6.
4. Leblebicioglu H, Ozaras R, Irmak H, Sencan I. Crimean-Congo hemorrhagic fever in Turkey: Current status and future challenges. Antiviral Res. 2016;126: 21–34.
5. Inci A, Ica A, Yildirim A, Vatansever Z, Cakmak A, Albasan H, et al. Economical impact of tropical theileriosis in the Cappadocia region of Turkey. Parasitol Res. 2007;101 (Suppl 2): S171-4.

**Key Learning Points**

- The geographic location of Turkey serves as a natural bridge for transmission of many emerging or re-emerging diseases among the continents of Europe, Asia, and Africa.
- Several argasid (genera *Argas*, *Ornithodorus*) and ixodid tick species (genera *Dermacenter*, *Hyalomma*, *Haemaphysalis*, *Ixodes*, and *Rhipicephalus*) have been responsable for the transmission of TBPs.
- Until today, 19 TBDs have already been reported in Turkey with a high economical impact.
- New disease management strategies and control programs against ticks and tick-borne infections should be put into practice based on the “One Health” concept in Turkey.
